# Improvement of Membrane Distillation Using PVDF Membrane Incorporated with TiO_2_ Modified by Silane and Optimization of Fabricating Conditions

**DOI:** 10.3390/membranes11020095

**Published:** 2021-01-29

**Authors:** Fida Tibi, Seong-Jik Park, Jeonghwan Kim

**Affiliations:** 1 Department of Environmental Engineering, Program in Environmental and Polymer Engineering, Inha University, Inharo 100, Michuholgu, Incheon 22212, Korea; tibifida7@gmail.com; 2 Department of Bioresources and Rural System Engineering, Hankyong National University, Anseong 17579, Korea; parkseongjik@hknu.ac.kr

**Keywords:** membrane distillation, ammonia rejection, permeate flux, silane, response surface methodology

## Abstract

The objectives in this study are to improve the performance of PVDF membrane by incorporating TiO_2_ and silane at various dosages and optimize fabricating conditions by using response surface methodology (RSM) for membrane distillation (MD) application. The PVDF membrane was synthesized by phase inversion method using various TiO_2_, silane and polymer concentrations. Membranes were characterized by performing contact angle measurements, SEM and FTIR observations. Ammonia rejection and permeate flux were measured by operating a direct contact distillation module treating ammonium chloride solution. A PVDF membrane created by adding TiO_2_ modified by silane improved membrane hydrophobicity. However, the effect of silane on membrane hydrophobicity was less pronounced at higher TiO_2_ concentrations. Highest ammonium rejection was associated with the highest membrane hydrophobicity. RSM analysis showed that fabricating conditions to achieve highest flux (10.10 L/m^2^·h) and ammonium rejection (100.0%) could be obtained at 31.3% silane, 2.50% TiO_2_, and 15.48% polymer concentrations. With a PVDF-TiO_2_ composite membrane for MD application, the effect of TiO_2_ was dependent upon silane concentration. Increasing silane concentration improved membrane hydrophobicity and ammonium rejection. RSM analysis was found to bea useful way to explore optimum fabricating conditions of membranes for the permeate flux and ammonium rejection in MD.

## 1. Introduction

Membrane distillation (MD) is a separation technology using temperature differences through a hydrophobic membrane. With MD membrane, separation occurs by the transport of water vapor, which is driven by the transmembrane temperature between feed solution and membrane permeate [1,2]. Operated at a very low pressure, the MD separation provides excellent permeate quality, requiring much low-fouling potential as compared to that observed with high pressure-driven membrane separation such as reverse osmosis (RO) membrane filtration [3,4]. With the advantages mentioned, MD membrane has been widely applied to remove salinity as an alternative to RO membrane for seawater desalination. However, not much information is available to understand the performance of an MD membrane for wastewater treatment applications. Inthis regard, membrane materials play a core role in determining the performance of MD membrane in wastewater treatment, but few research studies have been conducted to investigate and optimize the combined effect of fabricating conditions on it. Membrane materials designed for MD processesare often required to have a high porosity and low thermal conduction. Most importantly, intrinsic properties of membrane materials such as hydrophobicity are required to avoid the aqueous solution from its entering into a dry pore matrix. Membrane hydrophobicity can determine the extent to which pore wetting occurs; thus, the loss of membrane hydrophobicity can often lead to high mass transfer resistance against membrane due to the formation of membrane poresthat are filled by the liquid, thus subsequently deteriorating MD performance [5]. 

In recent years, the use of inorganic nanocomposite materials has expanded significantly with MD membrane to reduce membrane fouling. A range of nanoparticles such as SiO_2_, ZnO and TiO_2_ [1] have been added to various polymeric membranes as shown in Table 1. Among them, TiO_2_ has been paid much attention because of its wide availability and high physicochemical properties, as well as its antifouling capabilities [1,2,3]. Membranes applied in MD process consists mainly of hydrophobic characteristics by using appropriate polymers such as polypropylene (PP), polytetrafluoroethylene (PTFE) and polyvinylidene fluoride (PVDF) [4], as also summarized in Table 1. Among them, PVDF is preferable as aphase separation method to synthesize the MD membrane due to high solubility in various solvents and wide application in the MD process [5].

Nevertheless, polymeric membranes for the MD process are inherently prone to be fouled easily with pore wetting due to hydrophobic–hydrophobic interactions between organic contaminants and membrane materials [6,7,8,9]. Prior to applying inorganic additives to improve the performance of MD membrane, they are modified chemically by coupling the silane not only to improve membrane hydrophobicity but also preventing aggregation of nanoparticles embedded in the membrane matrix. The silane coupling is responsible for reducing the hydrophilic nature of inorganic particles and their surface energy [10,11]. Although the desired compound can be concentrated by the MD membrane effectively, its recovery can be reduced due to the loss of hydrophobicity of the membrane [10,11,12]. Therefore, maintaining membrane hydrophobicity should be core to determiningthe performance of MD membrane in wastewater treatment.

Although the importance of MD technology has been growing rapidly in wastewater treatment, the main obstacle in MD membranes for the treatment of a characteristic environmental pollutant is the rejection of inorganic contaminants, such as ammonium ions. However, few efforts have been made to optimize the fabricating conditions of membrane materials with respect to ammonium rejection in MD applications efficiently.Direct contact membrane distillation (DCMD) was applied as a post-treatment followed by the anaerobic membrane bioreactor (AnMBR), where ammonium removal efficiency is quite limited. As a commercial PVDF membrane was used, a previous study showed that about 76–94% of ammonium rejection efficiency could be obtained by using the permeate produced by an anaerobic membrane bioreactor (AnMBR) system treating low-strength wastewater [13]. 

Shortfalls of strategies to improve membrane materials have encouraged suggestions for further research in MD application. Shifting the hydrophobicity toward superhydrophobicity helps to introduce an air gap between liquid droplet and membrane surface [14]. This air gap provides an opportunity to increase the allowable pore sizes prior to the occurrence of pore wetting, consequently ensuring higher mass flux [15]. Coating with inorganic nanoparticles such as TiO_2_ or SiO_2_ on membrane surfaces has allowed the formation of hierarchical structure for superhydrophobic surfaces. Recently, fluorosilanized TiO_2_ nanoparticles were embedded in microporous PVDF membranes, displaying a hierarchical structure with multilevel roughness. The derived membrane showed superhydrophobicity with desirable anti-fouling properties [16]. Deposit of salt within membrane pores also decreased because the extent of partial wetting could be reduced by coating the TiO_2_ on the membrane [7]. On the other hand, MD membrane was studied for the treatment of silane in the presence of organic compound by using a modified TiO_2_-PVDF membrane. It was found that membrane hydrophobicity significantly contributed organic removal efficiency such as phenol compound [17].

In spite of many efforts to fabricate mixed matrix membranes for MD applications, the efficacy of silanized TiO_2_-embedded PVDF membranes on the rejection of inorganic pollutants such as ammonium still needs to be studied and optimized. Furthermore, membrane synthesis has been carried out by applying the conventional ways involving variation of one parameter while the others remain constant. This method often requires many experimental runs to find out optimum fabricating conditions, which needs a significant amount of time and chemicals [18,19]. Although the feasibility of TiO_2_-modified membranes has been studied for MD applications, optimizing fabricating conditions is needed to evaluate new membrane materials for inorganic rejection efficiency. 

In this work, PVDF membranes were fabricated by incorporating them with TiO_2_ nanoparticles coupled with 1H, 1H, 2H, 2H-heptadecafluorodecyl silane for improving membrane hydrophobicity in direct contact membrane distillation (DCMD) application to investigate combined effects of fabricating conditions on ammonium rejection and membrane permeability. To overcome the limitations of conventional methods to determine fabricating condition, response surface methodology (RSM), which is well-known as a statistical approach, was conducted in this study to optimize it by evaluating the Box-Behnken model design and analyzing experimental factors with small experimental runs. Three independent variables, including concentrations of TiO_2_, silane and polymer concentration, were selected. 

## 2. Materials and Methods 

### 2.1. Chemicals

PVDF powder was purchased from Alfa Aesar (Ward hill, MA, USA) and dried at 100 °C before being used. N-methyl-2-pyrrolidone (NMP) (>99.5%) (Daejung Chemicals and Metals, Gyeonggi-do, Korea) was used as the polymer solvent. TiO_2_ nanoparticles of 21 nm as primary particle size and higher than 99.5% trace metal basis for doping solution were acquired from Sigma-Aldrich (St. Louis, MO, USA). The other additives of the doping solution, including ortho-phosphoric acid (H_3_PO_4_) (>85%) and acetone, were purchased from Merck (Darmstadt, Germany). Ethanol was used as silanation solvent. The trichloro (1H, 1H, 2H, 2H-heptadecafluorodecyl) silane, which is acquired by TCI (Tokyo, Japan) was used in this study. For MD experiment, a 35 mg/L of ammonium chloride (NH_4_Cl, Duksan Pure Chemicals, Gyungggi-do, Korea) was prepared as a feed solution in deionized (DI) water. 

### 2.2. Synthesis and Modification of PVDF Membrane

Membranes were synthesized by phase inversion method according to the literature [17]. PVDF powder was dried at 100 °C for 24 h to remove its moisture content before preparing the doping solution. The dried PVDF at different concentrations was mixed with N-Methyl-2-Pyrorolidone (NMP) solvent and non-solvent additives including acetone (5 wt%) and H_3_PO_4_ (3 wt%) to improve the porous structure. The mixture of the solution was stirred for 24 h at 50 °C before degassing for another 24 h. The solution was cast on a polyester nonwoven fabric placed on a glass plate with a casting knife with 250 µm thickness (Elcometer) at room temperature. The casted film was immersed into a coagulation bath containing pure ethanol for 15 min. After this, the membrane was immersed into a second coagulation bath containing DI water for 24 h. The wet membrane was then dried in the oven at 50 °C for 72 h. For silane modification, silane was mixed with ethanol at various concentrations, and then it was stirred for 30 min. The TiO_2_ nanoparticles were added into the silane solution for 5 min and separated by 1.2 µm glass-fiber filtration. The TiO_2_ nanoparticles obtained were rinsed by using ethanol to remove residual silane and then dried in the oven at 50 °C for 24 h before being used in the dope solution. The membranes were characterized and tested by using the MD module for treating ammonium chloride solution. Fabrication conditions applied in this study are summarized in Table 2.

### 2.3. Membrane Characterization

The contact angle on the membrane surface was measured by using Contact Angle Analyzer (Surfatech, GSA-X, Vendargues, France) to investigate the change of membrane hydrophobicity. DI water was dropped through a syringe onto the membrane surface at room temperature. Replicate measurement was conducted at three different positions on the membrane surface. Liquid entry pressure (LEP) is a measurement of the ability of a hydrophobic membrane against pore wetting, and it can be obtained using a dead-end filtration cell (Sterlitech, Kent, WA, USA, HP4750). The reservoir was first filled with DI water, and then a dry membrane sample was tightly secured in the cell. The pressure was increased stepwise after a 5 min waiting period. The appearance of the first drop output was taken as a liquid entry pressure [10]. Membrane porosity, *ε* (%), was determined by a gravimetric method. A membrane sample with a weight of *W*1 (g) was immersed in ethanol for 2 h. After that, the sample was taken out and weighed after both of its sides were wiped off by absorbent cotton. Then, the weight of the wetted sample was measured again, W2 (g). W3 (g) was the weight of ethanol absorbed separately by the non-woven support layer. Equation (1) was used to determine the porosity, where *ρ* (g/cm^3^) is the density of ethanol, *A* (cm^2^) is the membrane area, δ1 (cm) is the total membrane thickness and δ2 (cm) is the non-woven support layer thickness. Five membrane samples were measured to obtain the average value.

(1) $$\varepsilon =\frac{\left(w2-w1-w3\right)/\rho }{\left(\delta 1-\delta 2\right)\times A}$$

Fresh and used membranes were observed by Fourier transform infrared spectroscopy (FT-IR) (Bruker VERTEX 80V, Billerica, MA, USA). Surface morphology of membranes was further observed using scanning electron microscopy (SEM) (SEM, FEI Inspect F50, Westborough, MA, USA) coupled with EDX in order to analyze the surface composition of the membrane. The permeate quality produced by the MD membrane was analyzed by employing the persulfate digestion to measure ammonia nitrogen using a reagent kit (Hach, 2672145, Loveland, CO, USA). Topography images of the membrane surfaces were obtained by using atomic force microscopy (AFM, NX-10, Park Systems, Jeju, Korea). The scanning area was XY (150 µm) and Z (5 µm), and the type of probe wasSi-N, working in tapping mode.

### 2.4. Experimental Set-Up ofDirect Contact Membrane Distillation 

**Figure 1:**
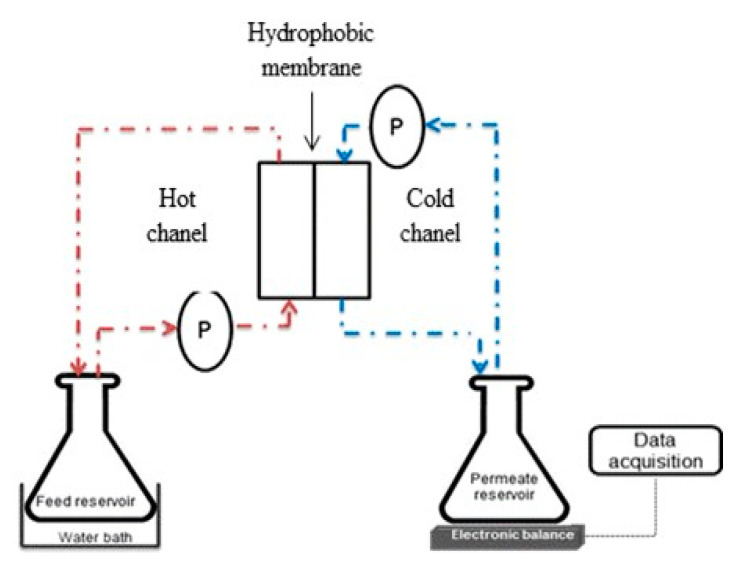
Experimental set-up of direct-contact membranedistillation(DCMD).

The MD filtration was performed by applying a laboratory-scale direct contact membrane distillation (DCMD) experimental set-up consisting of a membrane module and feed and permeate reservoirs as shown in Figure 1. The feed reservoir was placed into a water bath equipped with a temperature controller. The feed solution consisting of 35 mg/L ammonium chloride (NH_4_Cl) was heated by elevating the temperature to 60 °C through the water bath to allow only vapor across the membrane. The vapor formed from the membrane was then condensed by DI water filled in the permeate reservoir at constant temperature of 25 °C. Gear pumps (WT3000-1FA, Longerpump, China) were used to recirculate feed and permeate stream through water channels of MD membrane module at the same flow rate of 500 mL/min. The permeate flux of the membranes prepared, *J,* was calculated by the following equation

(2) $$J=\frac{∆m}{\rho \times A_{m}\times ∆t}$$

where *J* is the permeate flux (L/m^2^·h), m is the mass of permeate (kg), *A_m_* is the effective area of membrane (m^2^), *t* is the distillation time (h), and *ρ* is the density of permeate solution, which is 1 g/cm^3^. The rejection coefficient, R (%) was calculated according to the following equation:

(3) $$R=[1-\frac{C_{p}}{C_{F}}]\times 100$$

where *C_F_* is the concentration of the feed (g/L) and *C_p_* is the concentration of permeate (g/L). In addition, relative flux was determined to understand the flux decline pattern,

(4) $$\mathrm{Relative}\;\mathrm{flux}\;=\;\frac{J}{\mathrm{Jw}}$$

where *J* is the permeate flux measured with ammonium chloride in the feed and Jw is permeate flux measured with pure water as the feed.

### 2.5. RSM for Experimental Design and Optimization

RSM reduces process time and production cost to obtain the optimum condition of the membrane synthesis. Three independent variables (silane, TiO_2_ and polymer concentration) were considered for optimization, while the response variables were the permeate flux (L/m^2^·h) and ammonium rejection (%). Design-Expert statistical software (version 7.0.0. STAT-EASE Inc., Minneapolis, MN, USA) was used to compile the 17 sets of experimental conditions and analyze them based on the Box–Behnken model. The variables and levels selected for the study are shown in Table 3. The experimental data obtained based on the above design was fitted to a second-order polynomial equation. A quadratic model used for generated response surfaces is represented as follows:

(5) $$Y=a_{0}+a_{1}A+a_{2}B+a_{3}C+a_{12}AB+a_{13}AC+a_{23}BC+a_{11}A^{2}+a_{22}B^{2}+a_{33}C^{2}$$

where *Y*: the predicted permeate flux (L/m^2^·h) or ammonium rejection (%); *a*_0_: the constant coefficient; *a_i_*, *a_ii_* and *a_ii_*: the linear, quadratic, and interaction coefficients respectively; *A*: silane concentration (wt%); *B:* TiO_2_ concentration (wt%); and *C*: polymer concentration (wt%). The regression analysis, statistical significance and response surfaces were justified through analysis of variance (ANOVA).

## 3. Results and Discussion 

### 3.1. Membrane Porosity 

As shown in Table 2, at the same silane (25.7%) and TiO_2_ concentrations (1.5%), membrane porosity decreased from 62.1 to 50.4% when polymer concentration increased from 14 to 16 wt%.As silane concentration was reduced to 17.1% while TiO_2_ concentration increased to 2%, a similar result was observed, namely that increasing the polymer concentration reduced the membrane porosity (67.8% vs. 52.1%). In other aspects, porosity changes can be attributed to the fact that increasing polymer concentration suppresses macro-void structure because the exchange rate between solvent and non-solvent becomes faster. In addition, with a higher ratio of water concentration to polymer concentration, higher porosity of membrane can be formed [25]. 

**Figure 2:**
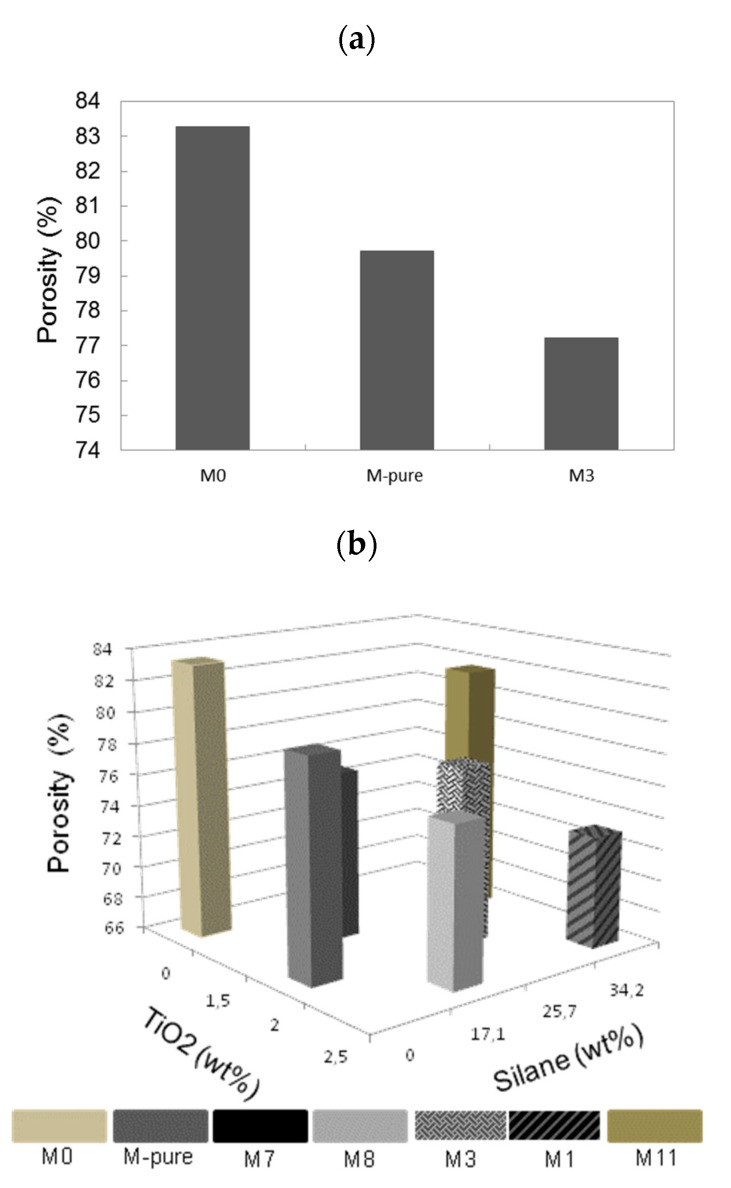
(**a**) The effect of TiO_2_ concentrationon membrane porosity and (**b**) combined effect of TiO_2_ and silane dosage on membrane porosity.

Figure 2a shows the effect of TiO_2_ and silane concentrations on membrane porosity. At 15 wt% of PVDF, M0 membrane without adding the silane and TiO_2_ achieved the highest membrane porosity of 83.2%. With ethanol as the first coagulation bath resulting in PVDF membranes with high porosity, demixing rate during the phase inversion can influence the formation of membrane pore size and porosity [26]. Nevertheless, adding the 2 wt% of TiO_2_ into the PVDF membrane enhanced thermodynamic instability, thus decreasing the effective time required for the growth of membrane pore structure, which later produced a membrane that was less porous at 79.7% porosity [27]. For both unmodified and modified TiO_2_–PVDF membranes, the porosity of PVDF membrane was reduced, probably due to the aggregation of TiO_2_ particles within membrane pore matrix to 79.7% and 77.2% for M-pure and M3, respectively (Supplementary Materials Figure S1). On the other hand, the membrane porosity was affected by TiO_2_ and silane concentration as shown in Figure 2b. At 15 wt% of PVDF and 17.1 wt% of silane concentration, increasing the TiO_2_ dosage from 1.5 to 2.5 wt% reduced the membrane porosity from 76.6 to 75.9% slightly probably due to a hindrance effect of nanoparticles during phase inversion process. In addition, under the same silane concentration (34.2 wt%) and polymer concentration (15 wt%), membrane porosity decreased from 81.6 to 73.0% as TiO_2_ concentration increased from 1.5 to 2.5 wt%. 

**Figure 3:**
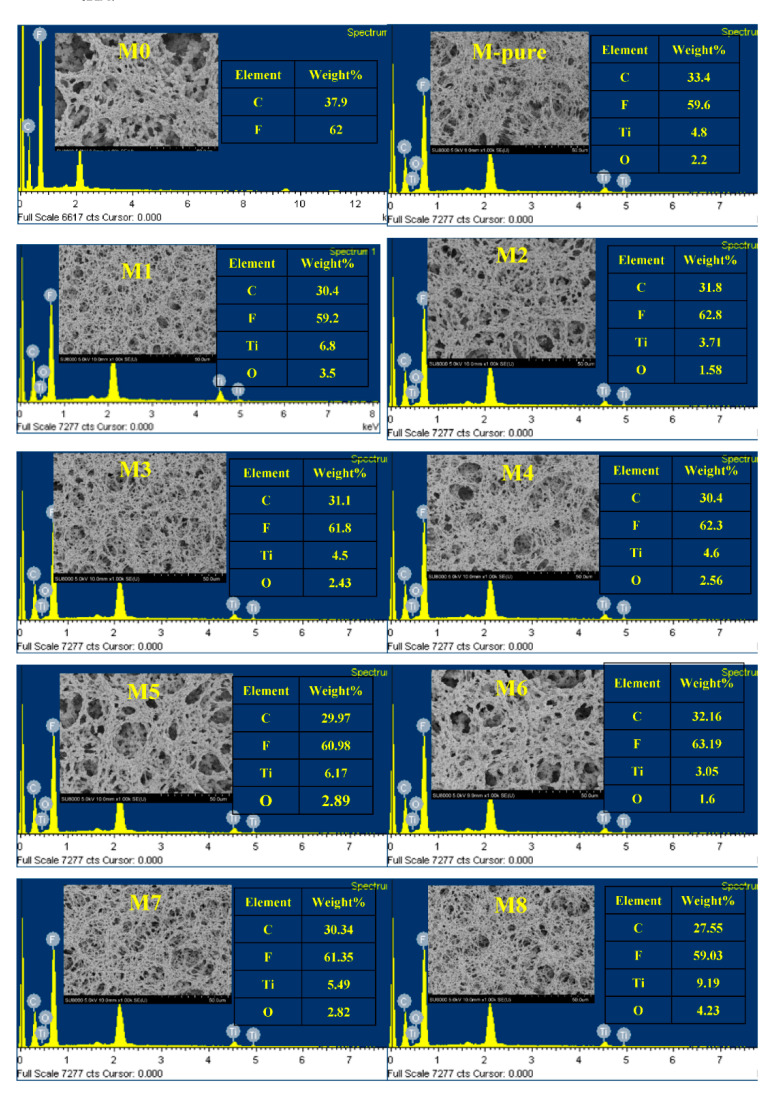

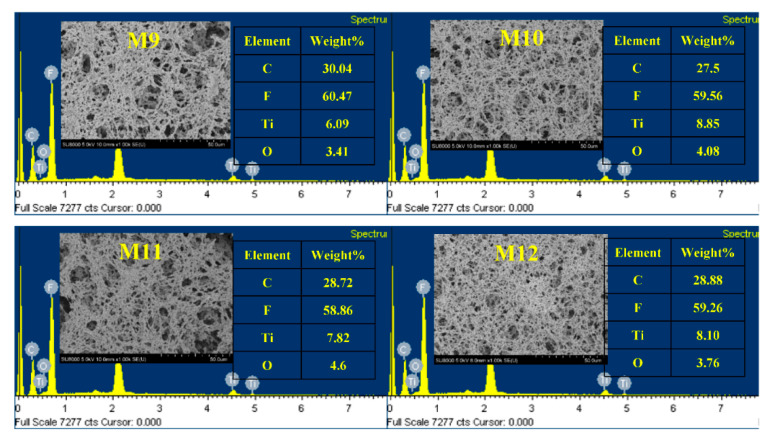
SEM-EDX analysis of PVDF membranes prepared at different polymer, TiO_2_ and silane concentrations.

**Figure 4:**
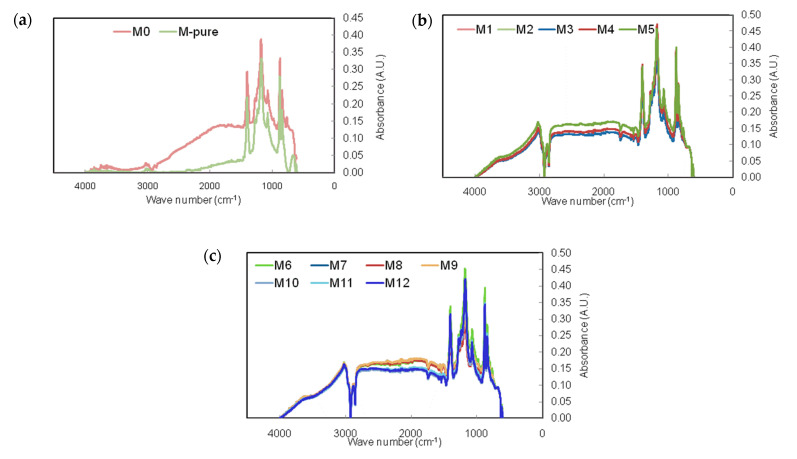
FT-IR spectra of the PVDF membranes before and after silane modification.

The SEM images of plain views of the membrane in Figure 3 demonstrate that surface morphology of PVDF membrane is similar across the whole membrane. It can be obviously seen that all the prepared membranes showed intrinsically similar features based on plain views by SEM throughout the membrane with an interconnected fibrous structure. We also used NMP wetted support, which is a suitable solvent for PVDF; therefore, the phase inversion can occur in the whole membrane surface, where neat films are cast on a glass plate. It can be seen that the increase in polymer concentration from 14 to 16 wt% exerted a slight effect on membrane surface morphology. Figure 3 also shows the results of SEM–EDX analysis with the elemental composition of the membrane surface. The presence of carbon (C), oxygen (O), fluorine (F) and titanium (Ti) elements detected in these EDX imagesrepresent PVDF polymer and TiO_2_ nanoparticles in both modified and unmodified membranes.Unlike FT-IR spectra in Figure 4, the existence of silane (Si) in membranes could not be detected by SEM images, so that it might have been embedded in the membrane matrix.

The FTIR spectra showed insignificant changes inPVDF membrane after modification by using different silane and TiO_2_ concentrations (Figure 4). The amorphous phase of PVDF membrane was represented by the peaks between 840 and 870 cm^−1^, while the peaks at 1072 cm^−1^ represent the crystal phase [28]. The bands between 800 and 650 cm^−1^ proved the existence of TiO_2_ in the membrane, as also reported by others [26,29]. 

However, the broad absorbance from 1174 to 1081 cm^−1^ indicates the formation of Si-O-Ti bonds (and perhaps Si-O-Si bonds). In the spectrum of modified membranes, the bands at 2883 and 3022 cm^−1^ are assigned to C-H stretching vibration, which corresponds to the presence of silane in modified membranes [28].The band appearing at 1402 cm^−1^ is due to the C-F stretching vibrations, which confirms that the fluorinated group is successfully grafted on the titania surface.

### 3.2. Membrane Hydrophobicity 

**Figure 5:**
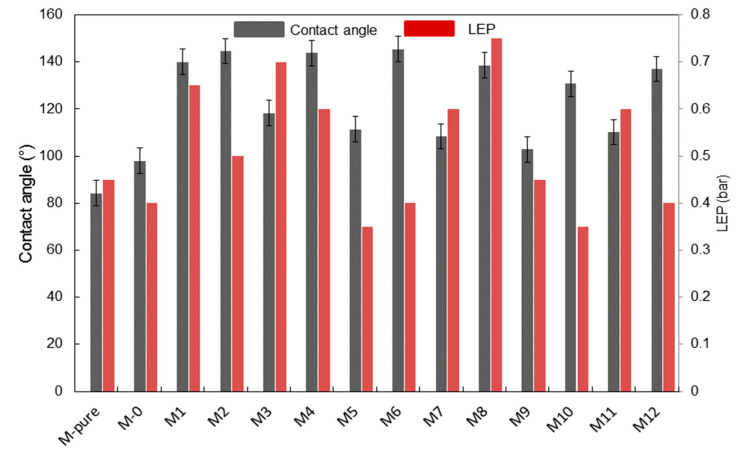
Contact angle of synthesized membranes.

The PVDF membranes were fabricated by introducing TiO_2_ at different concentrations. Prior to applying TiO_2_ nanoparticles, they were modified by adding the silane solution to improve membrane hydrophobicity as mentioned above [15]. Contact angle measurements were conducted for each TiO_2_ concentration. As a result of the high affinity of TiO_2_ nanoparticles to water, the addition of unmodified TiO_2_ without silane showed lower membrane hydrophobicity with 84.2° of water contact angle (M-pure) than bare PVDF membrane (97.9°) as shown in Figure 5.

**Figure 6:**
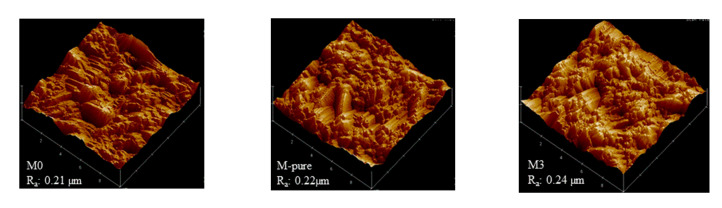
AFM images of M0, M-pure and M3 membranes.

However, hydrophobicity of M-pure could be achieved through the enhancement of surface roughness by adding TiO_2_ nanoparticles and using ethanol bath for phase inversions [15,16,30]. The surface roughness of this M0, M-pure and M3 was further analyzed using topography images generated by AFM instrument. Three-dimensional images in Figure 6 show that the addition of TiO_2_ affected surface roughness slightly, but the mean roughness of M3 increased after adding the fluoroalkylsilane.The MD membrane prepared by adding the unmodified TiO_2_ showed the lowest contact angle in this study. However, when adding the fluoroalkylsilane into TiO_2_ solution, the hydrophobicity of the membranes prepared was enhanced for all polymer and silane concentrations applied in this study, showing the range of contact angle from 100 to 145.5°(Figure 5). As compared to the contact angle of bare membrane prepared with 15% polymer and 2% TiO_2_ (M-Pure), adding the 25.7% of silane into TiO_2_ nanoparticles enhanced the membrane hydrophobicity by 118.3° of contact angle. Furthermore, the effect of TiO_2_ concentration on the hydrophobicity of the membrane was dependent on the concentration of silane. For two membranes (M11 and M1) prepared at 15 wt% of PVDF concentration and 34.2 wt% of silane concentration, results showed a contact angle of 130.5° and 140.4° for M11 and M1, respectively when increasing TiO_2_ concentration from 1.5 to 2.5 wt%. 

Figure 5 also shows results by contact angle measurements for different polymer concentrations. An increasing polymer concentration increased the contact angle of the membrane surface, supporting the idea that membranes became more hydrophobic at the same silane and TiO_2_ concentration (M10 and M12). In addition, membrane thickness was increased from 154.6 to 171 µm. This was caused by increased solution viscosity with increasing polymer concentration, which can hinder the penetration of nonsolvent through membrane pore during precipitation step [30,31]. Modified membranes such as M9 and M12 using 2 wt% of TiO_2_ and 16 wt% of PVDF showed that contact angle increased from 102.8° for M9 to 137.06° for M12, respectively as the silane concentration increased from 17.1 to 34.2 wt%. A similar result was found when comparing the M7 to M11, M8 to M1 and M5 to M10, namely that the contact angle increased with increasing the silane concentration from 17.1 to 34.2 wt% regardless of TiO_2_ and polymer concentrations. To know the change of contact angle of the membrane after MD experiment, the M1 membrane was selected. It was found that the contact angle was decreased from 140 to 120° after the experiment. More work is still needed to maintain high hydrophobicity of membrane for long-term MD operation.

**Figure 7:**
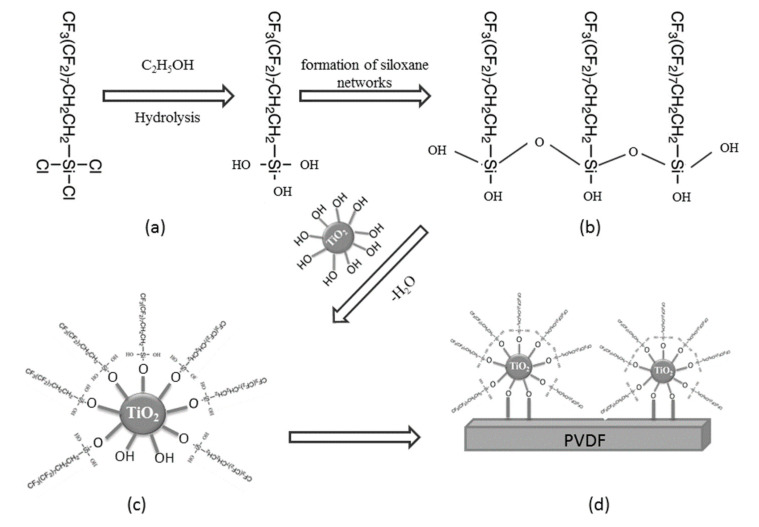
Schematic representation of silanization of the PVDF membranes: (**a**) hydrolysationof trichloro (1H, 1H, 2H, 2H-heptadecafluorodecyl) silane; (**b**) condensation and formation of siloxane networks via covalent bonding; (**c**) interaction with the surface of TiO_2_; (**d**) incorporation of modified TiO_2_ in PVDF dope solution.

The silane with fluorinated chains lowers the surface tension of membranes and creates a non-sticking property on membrane surface [32]. In ethanol as used in this study, the silane is hydrolyzed, and hydroxyl groups are formed. Fluorination mainly occurred on the active sites of TiO_2_ nanoparticles, involving the creation of covalent bonding between hydrolyzed silane and hydroxyl group (OH) of TiO_2_ nanoparticles within PVDF membrane matrix during the condensation period to allow siloxane networks (Si-O-Si) [16]. The hydroxyl groups in silane can from hydrogen bonding on the active sites of TiO_2_ nanoparticles where covalent bonding of Si-O-Ti occurs. Finally, TiO-silane complexes are entrapped in the PVDF membrane matrix as shown in Figure 7. This mechanism has been proposed for PVDF-TiO_2_ composite membrane [16], but more studies are still needed to better understand the formation mechanisms of PVDF membrane entrapped withTiO and silane at various fabricating conditions for MD application. The LEP_w_ values of all modified membranes obtained were retained near to the LEP_w_ of the unmodified membrane. The LEP_w_ of these membranes was not significantly affected by adding TiO_2_ and silane concentrations but was affected probably by the changes of pore shape besides surface hydrophobicity and pore size as shown in Figure 5 [33].

### 3.3. Permeate Flux and Ammonium Rejection 

**Figure 8:**
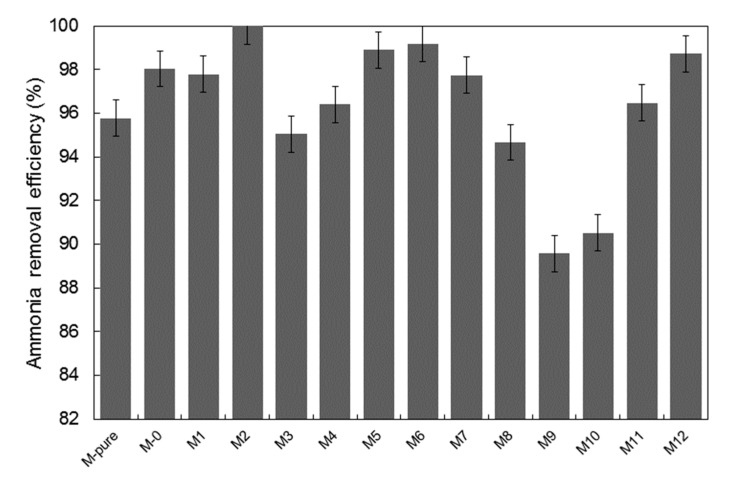
Removal efficiency of ammonium nitrogen of membranes synthesized.

**Figure 9:**
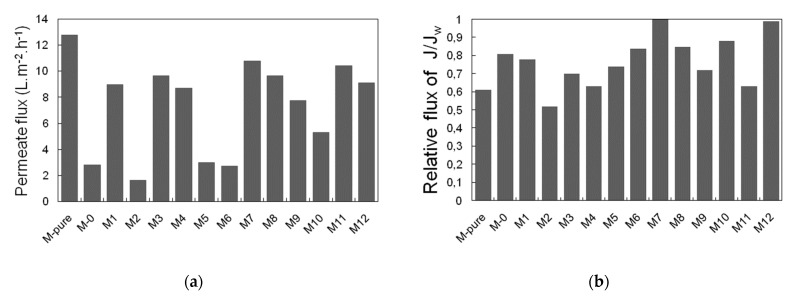
(**a**) Permeate flux variation of synthesized membrane treating ammonia chloride as feed solution and (**b**) relative flux of membrane after filtration.

Membrane efficiency in MD operation was evaluated in terms of ammonium rejection and permeate flux. Results are shown in Figure 8 and Figure 9. Despite having a difference in surface membrane characteristics, all the membranes in this study were able to retain 99.9% of ammonium ion. This can be attributed to the fact that the surface hydrophobicity was enhanced, as discussed above, which is crucial to prevent membrane wetting [16]. The variation in membrane porosity seems to affect the ammonium rejection insignificantly, as the surface hydrophobicity may be sufficient enough to prevent ammonia vapor from passing through the membrane to the permeate side. Additionally, higher ammonium rejection could be explained by the cation-exchange capacity of TiO_2_ to bind the ammonium ion present in feed solution [34,35,36]. It was reported that the titanate-based materials functioned as adsorbent for the removal of ammonium ion due to the interaction between Ti-OH and NH_4_^+^, which probably formed the hydrogen bonding [28]. Compared to our previous work, when treating the permeate produced by the staged anaerobic fluidized membrane bioreactor by using a commercial PVDF membrane, the ammonium rejection was around 85%, and this was lower than ammonium rejection achieved by TiO_2_-PVDF composite membranes fabricated in this study [13].

Figure 9 shows permeate flux and relative flux of each membrane synthesized in this study. In Figure 9a, M-pure membrane added with TiO_2_ only showed higher permeate flux (12.7 L/m^2^ h) than M0 membrane due to the hydrophilic characteristics of TiO_2_ [37]. Despite the high porosity of M0 and M5, they provided low permeate flux of 2.83 and 2.99 L/m^2^ h, probably due to relatively low thickness of these membranes, showing 153 and 156.5 µm, respectively. This is can be explained by the heat loss due to conduction, which tends to decrease the evaporation driving force, thus compromising vapor flux [38]. On the other hand, the membranes prepared at low PVDF concentrations (14 wt%) such as M2, M6 and M10 showed a stable permeate flux ranging 2–5 L/m^2^ h as compared to the membranes at relatively high PVDF concentration. This can be attributed to the fact that great hydrophobicity induces the formation of less porous structure and prevention of membrane wetting, since the ammonium is not detected in the permeate side [17]. 

The results of relative flux of each membrane are also shown in Figure 9b. Relative flux represents here the ratio of permeate flux for feed containing pure water to permeate flux for feed containing ammonium chloride after 6 h filtration. Relative flux also represents fouling resistance of the membrane to the feed solution, depending on membrane characteristics and solution composition, which lead to pore wetting and blocking [39]. Most of the membranes showed relative flux within the range of 0.7–1. Nevertheless, the relative flux of M-pure, M2, M4 and M11 was lower than 0.7. This could be related to increment of solute, concentration polarization, temperature polarization and wetting phenomena [23]. Although membrane fouling was observed by using the membranes synthesized in this study, the membrane wetting was not observed significantly after 6 h filtration as very low ammonium concentration was detected in permeate at higher than 90% of rejection efficiency through MD filtration. Therefore, membrane fouling that occurred during the experimental period was thought to contribute to flux decline. 

### 3.4. Application of Response Surface Methodology for Membrane Fabrication

The Box–Behnken model of RSM was employed in these experiments to obtain a quadratic model from 17 sets of experimental runs. The ranges and levels of the three independent variables, i.e., silane, TiO_2_ and polymer concentrations, are shown in Table 3. *F*-value tests were performed using an analysis of variance (ANOVA) to calculate the significance of each model employed in the design of expert statistical software. The results for permeate flux (L/m^2^·h) and ammonium rejection (%) recommended a quadratic model as the highest order polynomial model that satisfies that the model not be aliased. The experimental results obtained for permeate flux (L/m^2^·h) and ammonium rejection (%) were fitted with the quadratic model, which is represented by Equations (6) and (7), respectively, in terms of coded factors:

*Y_PF_* (L/m^2^·h) = 10.25 + 0.20*A*− 0.08*B* + 2.92*C* − 0.33*AB* − 0.25*AC* + 0.17*BC* − 0.07*A*^2^ − 0.47*B*^2^ − 3.90*C*^2^ (6)

*Y_AR_* (%) = 95.91 + 0.33*A* − 0.04*B* − 0.65*C* + 1.10*AB* + 4.38*AC* + 0.77*BC* − 1.69*A*^2^ + 2.44*B*^2^ + 0.21*C*^2^ (7)

**Figure 10:**
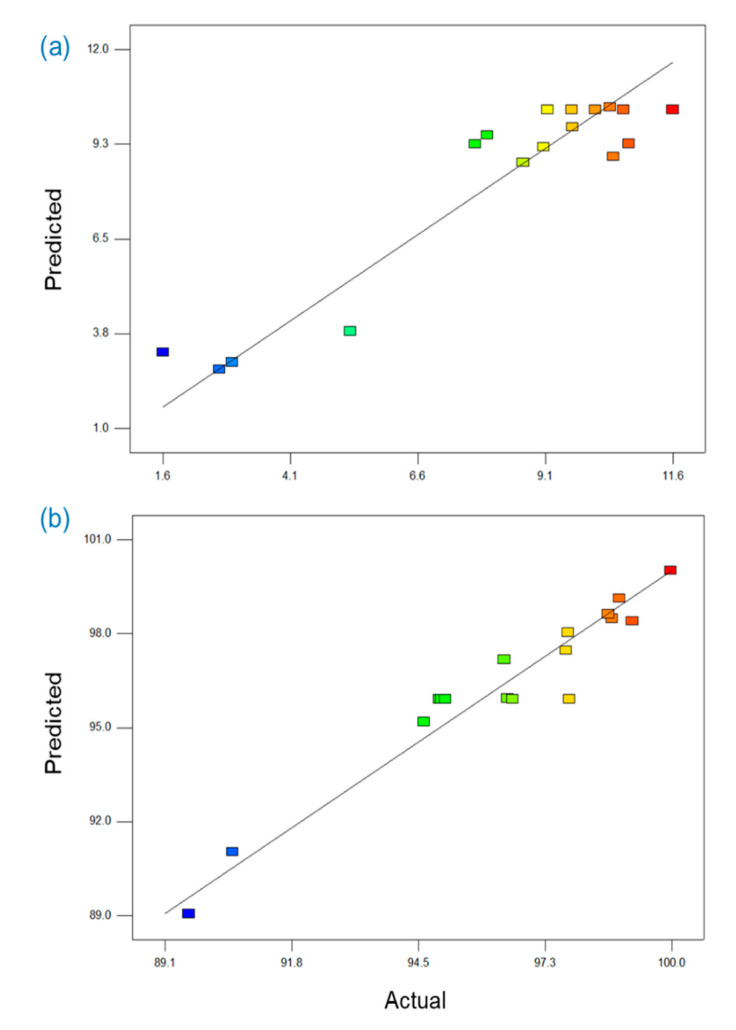
Plot of predicted values versus actual values for (**a**) permeate flux (L/m^2^·h) and (**b**) ammonium rejection (%).

The significance of the values of the model equations for permeate flux and ammonium rejection were checked by *F*, *R*^2^, adjusted *R*^2^, lack-of-fitand adequate precision tests. In the case of the permeate flux, as shown in Table 4, the model *F*-value, which was calculated by dividing the mean squares of each variable effect by the mean square, was 5.972, and the low probability value (0.0140), which was less than *p*-value at the 95% confidence limit, verified that the model terms were significant. The model probability value (0.0023) for ammonium rejection was also low enough to confirm that the model terms for ammonium rejection were significant. The goodness-of-fit of each model was also tested via the correlation coefficient *R*^2^, and the *R*^2^ values for the permeate flux and ammonium rejection were 0.885 and 0.934, respectively, indicating that both were reasonably close to 1 and thus acceptable. The adjusted *R*^2^ values for permeate flux and ammonium rejection were 0.737 and 0.848, respectively, and both values were statistically reasonable. Lack-of-fit tests were also used to evaluate the model adequacy; an insignificant lack-of-fit is desired [19,40]. The lack-of-fit values for permeate flux and ammonium rejection were 0.731 and 0.680, respectively; these values were statistically insignificant and showed that the constructed models were suitable to describe the observed data. The values of adequate precision, which reflected the signal-to-noise ratio, were 6.24 and 12.74 for permeate flux and ammonium rejection, respectively; because these values were greater than 4, they indicated adequate signals. Figure 10 shows that the points of the predicted versus actual plots for permeate flux and ammonium rejection are clustered along a diagonal line, indicating that the predicted values match well with the observed ones.

**Figure 11:**
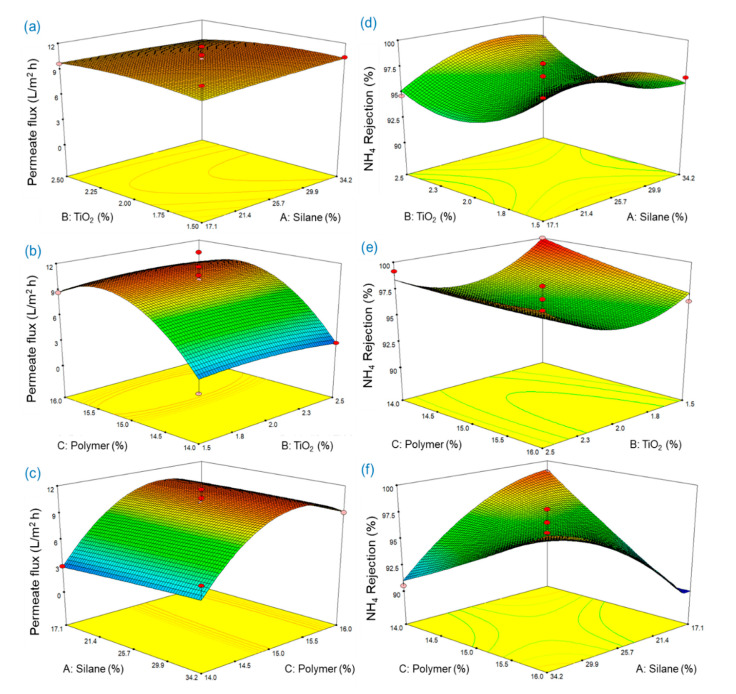
Estimated response surface for permeate flux (L/m^2^·h) showing the influence of (**a**) silane and TiO_2_ concentrations, (**b**) TiO_2_ and polymer concentrations and (**c**) polymer and silane concentrations. Estimated response surface for ammonium rejection (%) showing the influence of (**d**) silane and TiO_2_ concentrations, (**e**) TiO_2_ and polymer concentrations and (**f**) polymer and silane.

Table 4 shows that the significance of each term was determined by a *p*-value (Prob > *F*) less than 0.05 and that the linear and quadratic of polymer concentration significantly influenced the permeate flux. In Equation (6), a positive value for *C* indicates that the increase of polymer concentration has a positive effect on the permeate flux. However, a negative coefficient of *C*^2^ indicates that the permeate flux became lower at a higher polymer concentration. As shown in Figure 11, the permeate flux was increased as the polymer concentration increasedfrom 14.0 to 15.5%, but it was slightly decreased as the polymer concentration increased from 15.5 to 16.0%. In the case of ammonium rejection, *AC*, *A*^2^, and *B*^2^ were found to be significant factors influencing on the ammonia rejection of the synthesized membrane in this study. The interactive effects of silane and polymer concentrations on the ammonium rejection weresignificant, and the positive coefficient of *AC* indicated that the ammonium rejection was proportional to the product of silane and polymer concentration. The *p*-values of *A*^2^ and *B*^2^ were significant, but those of *A* and *B* were insignificant, indicating that the impact of silane and TiO_2_ at its highest value should be significant. The *p*-values more than 0.05 for *C* and *C*^2^ showed that the influence of polymer concentration on ammonium rejection was not statistically significant, which was in contrast to the results for permeate flux. 

To obtain optimum conditions, the desired values for each input and response factor can be selected using numerical optimization. The optimum conditions to maximize the responses, i.e., permeate flux and ammonium rejection, were calculated by statistical software. The highest permeate flux of 10.91 L/m^2^·h was obtained at 34.2% of silane concentration, 1.81% of TiO_2_ concentration and 15.33% of polymer concentration, and the maximum ammonium rejection of the synthesized membrane obtained from the RSM model was 100.4% at 24.8% of silane concentration, 1.50% of TiO_2_ concentration, and 14.01% of polymer concentration. Maximized permeate flux and ammonium rejection simultaneously were 10.10 L/m^2^·h and 100.0%, respectively, which were not significantly different from those obtained by optimizing each value. The conditions for obtaining both highest permeate flux and highest ammonium rejection were 31.3% of silane concentration, 2.50% of TiO_2_ concentration, and 15.48% of polymer concentration.

## 4. Conclusions

PVDF membranes were successfully synthesized by embedding TiO_2_ modified by silane to improve ammonia rejection and permeate flux in MD application. Membrane porosity decreased with increasing polymer concentration. Embedding TiO_2_ into the PVDF membrane improved hydrophilicity, but this effectiveness was less pronounced as silane concentration was higher. Increasing silane concentration enhanced membrane hydrophobicity but also reduced membrane porosity. The membrane synthesized provided the permeate flux ranging from 5.3 to 10.8 L/m^2^·h, with more than 90% of ammonia rejection efficiency. Highest ammonium rejection was associated with the highest membrane hydrophobicity since membrane wetting could be prevented by hydrophobic membrane surface by adding the TiO_2_ and silane. RSM analysis was found to be a useful way to determine optimized conditions to fabricate the PVDF-TiO_2_ composite membrane for enhancing both ammonia rejection efficiency and permeate flux in MD filtration. Due to the high retention propensity of MD membrane synthesized with inorganic contaminant such as ammonium ion as observed in this study, integrating anaerobic membrane bioreactor where nitrogen removal is very limited with an MD process may be proposed for energy-saving, wastewater reclamation. It can be also proposed that the nutrients produced by the anaerobic process can be concentrated directly by the MD membrane for future resource recovery.

## Figures and Tables

**Table 1:** Membrane properties and processing parameters for MD application in previous studies.

Membrane	Properties	Processing Parameters	Preparation	Ref
PVDF membrane with superhydrophobic TiO_2_	θ:160 ε:38–46; d:0.34–0.44	Feed: 100 g/LGA + 50 g/L NaCl; T_f_: 40 °C, T_p_: 20°C, Q: 300 mL/min	Phase Inversion	[17]
PVDF membrane with superhydrophobic SiO_2_	θ:139 ± 5 d:0.45	Feed: 3.5 wt%NaCl T_f_: 27.5°C, T_p_:15°C, Q: 2000 mL/min	Electrospinning	[20]
PVDF membrane with SiO2	θ:153 ε:79.24; d:0.1	Feed: 3.5 wt%NaCl T_f_: 70 °C, T_p_: 15 °C; Q: 3000 mL/min	Phase Inversion	[21]
PP membrane with SiO2	θ:157 ε:47.63	Feed: 3.5 wt%NaCl; T_f_: 80 °C	Phase Inversion	[22]
PVDFmembranewith superhydrophobic TiO_2_	θ:151.9 ± 1.5 ε:30.3 ± 3.9; d:0.37	Feed: 100 g/LGA + 50 g/L NaCl; T_f_: 40 °C, T_p_: 20; Q: 300 mL/min	Phase Inversion	[23]
PVDF membrane with superhydrophobic TiO_2_	θ:140 ± 2° ε:73%	Feed: 35 mg/L NH4Cl; T_f_: 60 °C, T_p_: 25 °C; Q: 500 mL/min	Phase Inversion	This study
Commercial PE membrane	θ: 108.3 ± 3 ε: 66.1 ± 7.2; d: 0.2	Feed: 3.5 wt% NaCl; T_f_: 80 °C, T_p_:17 °C;Q: 400 mL/min	Phase Inversion by TIPS	[24]
Commercial PVDF membrane	θ:111 d:0.22	Feed: AFMBR permeate (35 mg/L TN + 27 mg/L COD) T_f_: 60 °C, T_p_: 25 °C; Q: 500 mL/min		[13]

Note: θ: Contact angle (°); ε:Porosity (%); d: pore size (µm); T_f_: Feed temperature (°C); T_p_: Permeate temperature (°C); Q: Flow rate (mL/min).

**Table 2:** Combinations of fabricating conditions for PVDF composite membrane.

Membranes	Silane (w/w%)	TiO_2_ (wt%)	PVDF (wt%)	Porosity (%)	Contact Angle (°)	Thickness (µm)
M-pure	0	2	15	79.7	84.2	159.0
M0	0	0	15	83.2	97.9	153.0
M1	34.2	2.5	15	73.0	140.0	155.0
M2	25.65	1.5	14	62.1	144.6	159.3
M3	25.65	2	15	77.2	118.2	173.5
M4	25.65	1.5	16	66.8	143.8	164.0
M5	17.1	2	14	67.8	111.4	156.5
M6	25.65	2.5	14	66.3	145.4	146.3
M7	17.1	1.5	15	76.6	108.4	155.0
M8	17.1	2.5	15	75.9	138.5	149.3
M9	17.1	2	16	52.1	102.8	170.3
M10	34.2	2	14	64.4	130.7	154.6
M11	34.2	1.5	15	81.7	130.5	159.3
M12	34.2	2	16	63.8	137.0	171.0

**Table 3:** Coded and actual values of variables for the experiment.

Variables	Symbol	Coded Factor Level		
		−1	0	1
Silane (wt%)	A	17.1	25.65	34.2
TiO_2_ (wt%)	B	1.5	2.0	2.5
Polymer (wt%)	C	14	15	16

**Table 4:** Summary of ANOVA for response surface quadratic model of permeate flux and ammonium rejection. Underline indicates that *p*-value is less than 0.05.

Source	Permeate Flux					Ammonium Rejection				
	Sum of Squares	df	Mean Square	*F* Value	*p*-Value Prob > *F*	Sum of Squares	df	Mean Square	*F* Value	*p*-Value Prob > *F*
Model	135.65	9	15.07	5.972	0.0140	123.89	9	13.77	10.94	0.0023
*A*-Silane	0.33	1	0.33	0.130	0.7291	0.88	1	0.88	0.70	0.4312
*B*-TiO_2_	0.05	1	0.05	0.020	0.8908	0.01	1	0.01	0.01	0.9177
*C*-Polymer	68.21	1	68.21	27.027	0.0013	3.42	1	3.42	2.72	0.1433
*AB*	0.42	1	0.42	0.167	0.6947	4.80	1	4.80	3.81	0.0918
*AC*	0.24	1	0.24	0.095	0.7667	76.83	1	76.83	61.06	0.0001
*BC*	0.11	1	0.11	0.043	0.8414	2.37	1	2.37	1.88	0.2121
*A* ^2^	0.02	1	0.02	0.007	0.9335	12.01	1	12.01	9.54	0.0176
*B* ^2^	0.94	1	0.94	0.372	0.5614	25.04	1	25.04	19.90	0.0029
*C* ^2^	63.94	1	63.94	25.336	0.0015	0.18	1	0.18	0.14	0.7171
Residual	17.67	7	2.52			8.81	7	1.26		
Lack-of-fit	14.05	3	4.68	5.177	0.0731	2.54	3	0.85	0.54	0.6799
Pure Error	3.62	4	0.90			6.27	4	1.57		
Cor Total	153.31	16				132.69	16			

## Supplementary Materials

The following are available online at https://www.mdpi.com/2077-0375/11/2/95/s1, Figure S1: SEM images of PVDF membranes prepared at different polymer, TiO_2_ and silane concentrations.
