# Supplementary material for: Improvement of Membrane Distillation Using PVDF Membrane Incorporated with TiO2 Modified by Silane and Optimization of Fabricating Conditions
Source: Membranes (Basel). 2021 Jan 29;11(2):95. doi: 10.3390/membranes11020095 (PMC7912162; doi:10.3390/membranes11020095)
Supplement: Supplementary file 1 [file membranes-11-00095-s001.pdf]

Improvement of membrane distillation using PVDF membrane incorporated with TiO_2 modified by silane and optimization of fabricating conditions

FidaTibi[1], Seong-Jik Park[2] and Jeonghwan Kim[1,*]

[1] Department of Environmental Engineering, Program in Environmental and Polymer Engineering, Inha University, Inharo 100, Michuholgu, Incheon, Republic of Korea; tibifida7@gmail.com

[2] Department of Bioresources and Rural System Engineering, Hankyong National University, Anseong 17579, Korea; parkseongjik@hknu.ac.kr

* Correspondence: jeonghwankim@inha.ac.kr; Tel.: 82-32-860-7502

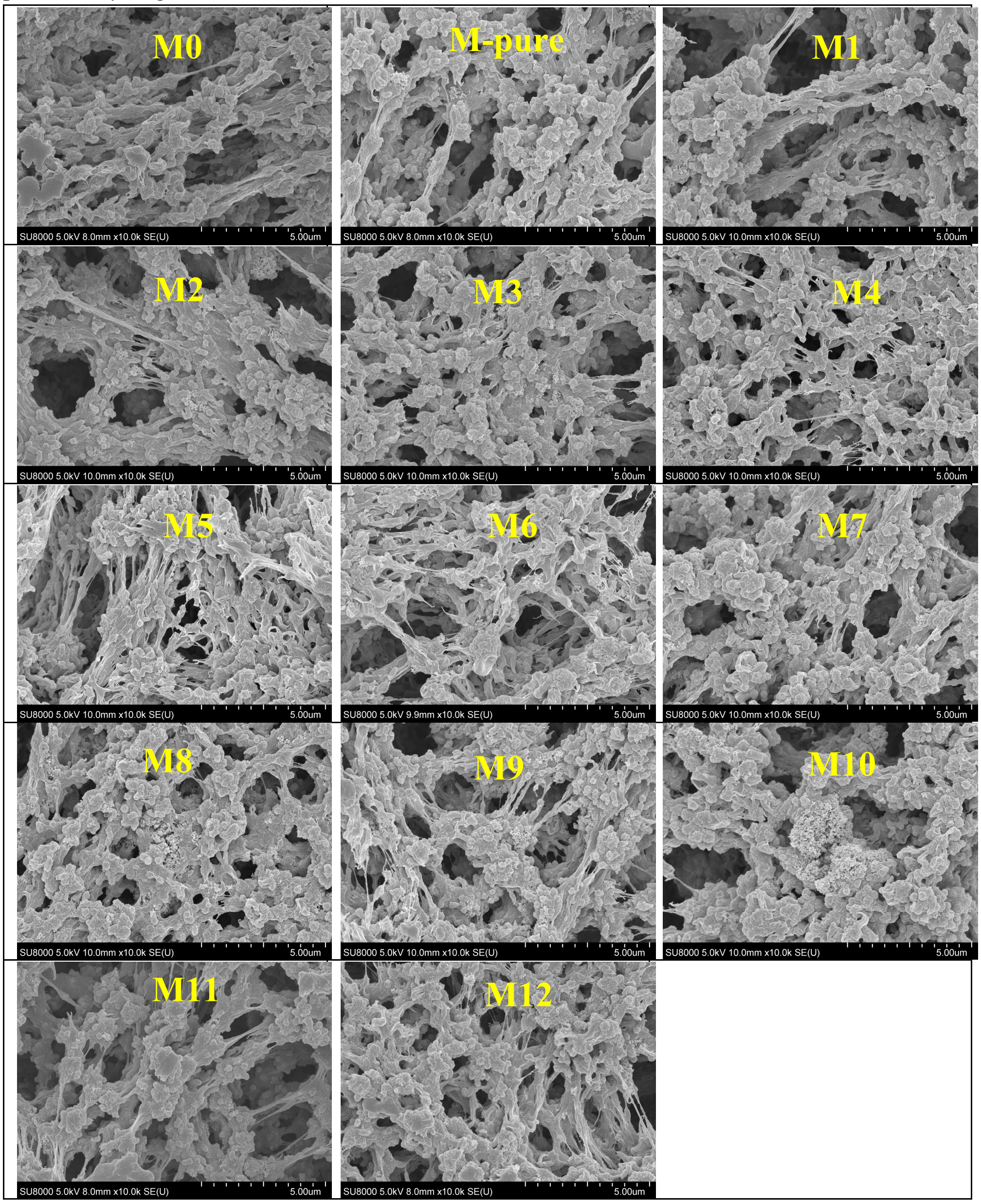

Figure S1: SEM images of PVDF membranes prepared at different polymer, TiO_2 and silane concentrations.
